# Canadian Partnership for Quality Radiotherapy (CPQR) and the Canadian Organization of Medical Physicists (COMP) — Driving safety and quality assurance practice in Canada through the development of technical quality control guidelines

**DOI:** 10.1120/jacmp.v17i6.6507

**Published:** 2016-09-08

**Authors:** Jean‐Pierre Bissonnette, Michael Milosevic, Marco Carlone, Kyle E. Malkoske

**Affiliations:** ^1^ Department of Radiation Oncology University of Toronto Toronto ON; ^2^ Techna Institute Toronto ON; ^3^ Princess Margaret Cancer Centre Toronto ON; ^4^ Canadian Partnership for Quality Radiotherapy Red Deer AB; ^5^ Canadian Organization of Medical Physicists Kanata ON; ^6^ Regional Cancer Program Royal Victoria Regional Health Centre Barrie ON Canada

Dear Editor

Cancer has been the leading cause of mortality in Canada this millennium, and it is anticipated that the rate with which it is diagnosed will increase as society ages. In 2015, 196,900 Canadians were diagnosed with cancer and 78,000 will die from the disease (http://www.cancer.ca/~/media/cancer.ca/CW/cancer%20information/cancer%20101/Canadian%20cancer%20statistics/Canadian‐Cancer‐Statistics‐2015‐EN.pdf?la=en). Almost half of all new cancer diagnoses will have radiation treatment prescribed at some point during their treatment journey.^(1)^ Today, there are 47 radiation treatment centers in Canada, most with academic affiliations.

The growing cancer burden, the increasingly complex interdisciplinary nature of cancer diagnosis and treatment, and the rapid technological evolution that has characterized our field highlight the need to put in place robust processes to ensure that high‐quality radiation treatment is delivered in a consistently safe way. In 2010, the Canadian Partnership for Quality Radiotherapy (CPQR) was founded as an alliance among the national professional organizations involved in the delivery of radiation treatment in Canada: the Canadian Association of Radiation Oncology (CARO), the Canadian Organization of Medical Physicists (COMP), and the Canadian Association of Medical Radiation Technologists (CAMRT), together with strategic and financial support from Health Canada through the Canadian Partnership Against Cancer (CPAC). CPQR was established to drive the development of system performance improvements in radiation treatment quality and safety. CPQR activities are centered around a partnership approach, and demonstrate a strong commitment to stakeholder engagement by ensuring that both the radiation treatment community and the patients are involved in the development, review, and validation of all programs and tools. This process facilitates broad uptake of new quality and safety programs within the radiation treatment community and a sense of ownership and commitment by front‐line staff, but also leadership support from provincial cancer agencies.

CPQRs successes to date have been far‐reaching. The Quality Assurance Guidelines for Canadian Radiation Treatment Programs (QRT Guideline, available at http://www.cpqr.ca/wp‐content/uploads/2013/09/QRT2015‐12‐03.pdf) was the seed document for the creation of Accreditation Canada's new Cancer Care Standards that will become part of the Qmentum accreditation process for Canadian hospitals in January 2017. The third iteration of the QRT Guideline was released in December 2015 and has seen broad adoption across the country. CPQR has also partnered with the Canadian Institute for Health Information (CIHI) on the development of a national system for reporting radiation treatment incidents. The system, which currently is being piloted at centers across the country, is structured to facilitate rapid dissemination of relevant incident information and discussion about ways to prevent incident recurrence and propagation. CPQR is committed to working with patients to ensure its programs are relevant to, and supported by, this community. It is poised to release the Patient Engagement Guidelines for Canadian Radiation Treatment Programs in June 2016 as a way to provide guidance for centers wishing to ensure appropriate patient and family engagement in issues related to quality and safety.

Early on, CPQR, together with COMP, identified the need to provide direction for assuming optimal performance of radiation treatment equipment. The earlier set of quality control guidelines prepared by the Canadian Association of Provincial Cancer Agencies was outdated, did not address the rapidly changing technologies, and had fallen into disuse. Working in collaboration, CPQR and COMP used the structured guideline development process common to all CPQR programs to develop "living quality control guidelines" that would meet the current and future needs of the Canadian medical physics community. The process incorporated expert review and revision, broad community consultation and, to assure relevance and practicality, comprehensive field‐testing with the intent to review and update the guidelines systematically every two years. The process is detailed in a paper published in this issue of the *Journal of Applied Clinical Medical Physics* (JACMP). This initiative has been a huge undertaking, involving more than 50 medical physicists from every Canadian province in the development and validation of the guidelines, and countless others in their review and field‐testing. The resulting suite of guidelines provides system descriptions and detailed daily, monthly, and annual quality control tests that should be incorporated into local quality assurance programs. Given the unprecedented level of peer‐review these guidelines have received, we intend to publish each guideline within the pages of the *JACMP*.

Moving forward, CPQR will continue to work with the medical physics community and the editors of the *JACMP* to support the ongoing review and evidence‐based revision of these guidelines to ensure that they remain useful tools to drive quality improvement. The two organizations will also work with international partners to promote integrated quality assurance programs where harmonization among countries can be invaluable in advancing radiation treatment practice.

## COPYRIGHT

This work is licensed under a Creative Commons Attribution 3.0 Unported License.
